# Entropy structural characterization of zeolites BCT and DFT with bond-wise scaled comparison

**DOI:** 10.1038/s41598-023-37931-2

**Published:** 2023-07-05

**Authors:** Micheal Arockiaraj, Daniel Paul, Muhammad Usman Ghani, Sushil Tigga, Yu-Ming Chu

**Affiliations:** 1 Department of Mathematics, Loyola College, Chennai, 600034 India; 2 Department of Mathematics, Sri Sairam Institute of Technology, Chennai, 600044 India; 3 Institute of Mathematics, Khawaja Fareed University of Engineering & Information Technology, Abu Dhabi Road, Rahim Yar Khan, 64200 Pakistan; 4 Department of Mathematics, Huzhou University, Huzhou, 313000 People’s Republic of China

**Keywords:** Chemistry, Materials science, Mathematics and computing

## Abstract

Entropy of a connected network is a quantitative measure from information theory that has triggered a plethora of research domains in molecular chemistry, biological sciences and computer programming due to its inherent capacity to explore the structural characteristics of complex molecular frameworks that have low structural symmetry as well as high diversity. The analysis of the structural order is greatly simplified through the topological indices based graph entropy metrics, which are then utilized to predict the structural features of molecular frameworks. This predictability has not only revolutionized the study of zeolitic frameworks but has also given rise to new generations of frameworks. We make a comparative study of two versatile framework topologies namely zeolites BCT and DFT, which have been widely utilized to create a new generation of frameworks known as metal organic frameworks. We discuss bond-additive topological indices and compute entropy measure descriptors for zeolites BCT and DFT using degree and degree-sum parameters. In addition, we perform bond-wise scaled comparative analysis between BCT and DFT which shows that zeolite BCT has greater entropy values compared to zeolite DFT.

## Introduction

Topological indices (TIs) are invariant molecular framework-related quantities that aid in framework characterization and the prediction of biological, chemical, and physical properties by establishing the relationship between the known properties of molecules and their topological indices. Such properties include boiling and melting points, temperatures of evaporation, acentric factor, density and tension of the liquid, pressure partition coefficient, etc. TIs have found several applications in reticular chemistry, drug designing, signal, image processing system and QSAR/ QSPR investigations^1–10^.

The topological indices can be generally classified into distance, degree, distance-degree and eigenvalue types indices. Although, there are numerous classification of indices, the indices based on degree have drawn uppermost attention of contemporary researchers due to their unique contributions in the arena of chemical graph theoretic investigations. The first degree based index known as Randić index was found to have a high degree of correlation with certain attributes of alkanes such as surface area, boiling points, enthalpy formation, chromatographic retention time and vapor pressure. Gutman and Trinajsti proposed Zagreb indices and applied them to study branching problems. They were extensively used to investigate heterosystems, chirality, ZE-isomerism and molecular complexity. Zhou and Trinajstic developed a sum connectivity index that exhibits a strong correlation with hydrocarbon $\pi$-electron energy. In this lineup, various types of degree based indices such as atom bond connectivity, harmonic, hyper-Zagreb, symmetric division degree, augmented Zagreb, geometric-arithmetic and Sombor indices have been introduced by highlighting diverse applications^11–20^. Furthermore, the analysis of the structural bias of the chemical structures of compounds can be accomplished using degree and degree-sum quantities^21–30^.

The number of unit distance vertices from $v_k$ is considered to be the degree, where $v_k$ is any vertex in a chemical graph *G* and it is represented by $d(v_k)$. A cluster of vertices that are located one unit distance from $v_k$ are said to be in the neighborhood of $v_k$. By adding the degrees in the neighborhood of $v_k$, we obtain the degree-sum of $v_k$ and it is represented by $d_s(v_k)$. We group the vertices of *G* in a set *V*(*G*), and describe two sets $D=\{d(v_k):v_k \in V(G) \}$ and $D_s=\{d_s(v_k):v_k \in V(G) \}$ respectively to present the vertex degree and degree-sum graph sequences. Again, we group the edges of *G* in a set *E*(*G*) and describe another two quantitative sets $$d_{(\alpha _k,\alpha _j)}= |\{{v_k}{v_j} \in E(G) : d(v_k) = \alpha _k \ $$ and $\ d(v_j) = \alpha _j \}|$ and $${d_{s}}_{(\alpha _k,\alpha _j)} = |\{{v_k}{v_j} \in E(G) : d_{s}(v_k) = \alpha _k \ $$ and $\ d_{s}(v_j) = \alpha _j \}|$ respectively to present the edge classes arising from degree and degree-sum. The additive degree and degree-sum categories of topological metrics are defined along with the multiplicative version of degree and degree-sum categories in the following for the index function $\zeta$.

$$\begin{aligned} {\zeta }^{d}(G)&= \underset{(\alpha _k,\alpha _j) \in D}{\sum }\ d_{(\alpha _k,\alpha _j)} \ \zeta (\alpha _k,\alpha _j)\\ {\zeta }^{d_s}(G)&= \underset{(\alpha _k,\alpha _j) \in D_s}{\sum } {d_s}_{(\alpha _k,\alpha _j)} \ \zeta (\alpha _k,\alpha _j)\\ {\zeta }^{d^{\times }}(G)&= \underset{(\alpha _k,\alpha _j) \in D}{\prod }\ {\zeta (\alpha _k,\alpha _j)}^{d_{(\alpha _k,\alpha _j)}}\\ {\zeta }^{{d^{\times }_s}}(G)&= \underset{(\alpha _k,\alpha _j) \in D_s}{\prod } {\zeta (\alpha _k,\alpha _j)}^{{d_s}_{(\alpha _k,\alpha _j)}} \end{aligned}$$

In our study, we discuss some special classes of index function that have recently received a lot of attention: First Zagreb $M_{1}(\alpha _k,\alpha _j) = \alpha _k+\alpha _j$, second Zagreb $M_{2}(\alpha _k,\alpha _j) = \alpha _k \alpha _j$, Randić $R(\alpha _k,\alpha _j) = \dfrac{1}{\sqrt{\alpha _k \alpha _j}}$, atom bond connectivity $ABC(\alpha _k,\alpha _j) = \sqrt{\dfrac{\alpha _k+\alpha _j-2}{\alpha _k \alpha _j}}$, harmonic $H(\alpha _k,\alpha _j) = \dfrac{2}{\alpha _k+\alpha _j}$, sum connectivity $SC(\alpha _k,\alpha _j) = \dfrac{1}{\sqrt{\alpha _k+\alpha _j}}$, hyper-Zagreb $HM(\alpha _k,\alpha _j) = \left( \alpha _k+\alpha _j\right) ^{2}$, geometric-arithmetic $GA(\alpha _k,\alpha _j) = 2 \dfrac{\sqrt{{\alpha _k \alpha _j}}}{\alpha _k+\alpha _j}$, irregularity $IRR(\alpha _k,\alpha _j) = \big |\alpha _k-\alpha _j\big |$, sigma $\sigma (\alpha _k,\alpha _j) = \left( \alpha _k-\alpha _j\right) ^{2}$, forgotten $F(\alpha _k,\alpha _j)= \alpha _k^2+\alpha _j^2$, Sombor $SO(\alpha _k,\alpha _j) = \sqrt{\alpha _k^2+\alpha _j^2}$, symmetric division degree $SDD(\alpha _k,\alpha _j) = \dfrac{\alpha _k}{\alpha _j}+\dfrac{\alpha _j}{\alpha _k}$, and augmented Zagreb $AZ(\alpha _k,\alpha _j) = {\left( \dfrac{\alpha _k \alpha _j}{\alpha _k+\alpha _j-2}\right) }^3$.

As we see from the description of the multiplicative topological indices, they grow rapidly when the size of the chemical structure increases which would be hard for chemists correlate these indices with chemical attributes. Therefore, we consider the reformulated multiplicative descriptors as explained in^29^, where the normal multiplication is converted to scalar multiplication. We now formally present the scalar multiplicative descriptors along with self-powered scalar multiplicative descriptors as below.

$$\begin{aligned} {\zeta }^{d^{*}}(G)&= \underset{(\alpha _k, \alpha _j) \in D}{\prod }\ {d_{(\alpha _k, \alpha _j)}} \ {\zeta (\alpha _k, \alpha _j)}\\ {\zeta }^{{d^{*}_s}}(G)&= \underset{(\alpha _k, \alpha _j) \in D_s}{\prod }\ {{d_s}_{(\alpha _k, \alpha _j)}} \ {\zeta (\alpha _k, \alpha _j)}\\ {\zeta }^{dp^{*}}(G)&= \underset{(\alpha _k, \alpha _j) \in D}{\prod } {d_{(\alpha _k, \alpha _j)}} \ {\zeta (\alpha _k, \alpha _j)}^{\zeta (\alpha _k, \alpha _j)}\\ {\zeta }^{{d_sp^{*}}}(G)&= \underset{(\alpha _k, \alpha _j) \in D_s}{\prod }\ {{d_s}_{(\alpha _k, \alpha _j)}} \ {\zeta (\alpha _k, \alpha _j)}^{\zeta (\alpha _k, \alpha _j)} \end{aligned}$$

The entropy of a connected network is a quantitative measure that is derived based on the information acquired from the structural properties to describe the order/disorder of networks, and this topic has gained remarkable recognition in recent years. Initially, the potential deliverables variable such as stochastic variable is considered to be the moderate measure of information. Let $p(x_i)$, $1 \le i \le n$, be the probability correspond to $x_i$ for the random variable *X*. The entropy of *X* is computed by the expresion $\displaystyle -\sum _{i=1}^{n} p(x_i)\log p(x_i)$ under probability informations as proposed by Shannon and denoted by *I*(*X*). Chemical molecules which are related to the topological descriptor informations $\zeta ^{\lambda }$, $\lambda \in \{d,d_s\}$, for which the entropy values^31,32^ are represented as follows:

1 $$\begin{aligned} I(\zeta ^{\lambda }(G))= & {} -\sum _{b\in E(G)} \frac{\zeta ^{\lambda }(b)}{\displaystyle \sum _{d\in E(G)}\zeta ^{\lambda }(d)} \log \left( \frac{\zeta ^{\lambda }(b)}{\displaystyle \sum _{d\in E(G)}\zeta ^{\lambda }(d)}\right) \nonumber \\= & {} \log \left( \sum _{b\in E(G)}\zeta ^{\lambda }(b)\right) -\frac{1}{\displaystyle \sum _{b\in E(G)}\zeta ^{\lambda }(b)} \log \left( \prod _{b\in E(G)}\zeta ^{\lambda }(b)^{\zeta ^{\lambda }(b)}\right) \end{aligned}$$

We change the normal multiplicative term in the above equation by scalar multiplicative term as discussed in the paper^29^, we arrive the modified Shannon’s entropy version as

2 $$\begin{aligned} I^*(\zeta ^{\lambda }(G))=\log \left( \zeta ^{\lambda }(G)\right) -\frac{1}{\zeta ^{\lambda }(G)} \log \left( \zeta ^{\lambda p^*}(G)\right) \end{aligned}$$

## Zeolites BCT & DFT

Zeolites are a class of nanomaterials basically consisting of SiO$_{4}$ or AlO$_{4}$ tetrahedral frameworks where recurring arrays of pores and channels give rise to large tuneable internal surface that allow the light hydrocarbons and water molecules to diffuse through them. The alluminosilicates have enormous potential for isomorphous substitution of Be, B, Fe, P, Ge, Co and Zn into the tetrahedral position of frameworks that greatly influence the structural characteristics of chemical compounds^33^. There have been strenuous efforts to discover new compositions and architectures particularly after the successful synthesis of microporous aluminoshosphates in 1982 and researchers have reported aluminophosphates, zincophosphates, iron phosphates and many other bimetallic phosphates in the field framework studies^34,35^. Thus, the incorporation of active metals into open-framework structures has given rise to many new structures analogous to zeolite topologies^36^. Zeolite DFT and BCT are two versatile framework topologies that arouse great interest among researchers due to their structural simillarities and tremendous potential to generate various types of novel frameworks.

Zeolite DFT derives its name and code from Davy Faraday number-2 (DAF-2), the first cobalt, phosphorous and oxygen-based framework. DFT framework topology can be described as the cross link of one dimensional bifurcated hexagonal squares. The adjustment of nodes in a four-ring can give various patterns of nets, which is common in the structures analogous to the DFT framework. DFT topology provides an interesting domain to explore the relationship between silicates and phosphates related framework topologies^36,37^. Zeolite BCT (body-centered tetragonal tectosilicate) framework is related to other tectosilicate. In the unit cell of the BCT framework, the four membered rings (4MRs) are interconnected into 6 and 8 MRs with two types of large cavity sites^38^. The existence of the laumontite cage surrounded by two pairs of opposite 6-rings and two opposite 4-rings is a crucial component of the BCT architecture^39^.

Zeolites DFT and BCT framework topologies consist of 4-connected open framework with TO$_{4}$ building blocks that forms a basis to construct metal organic frameworks (MOFs) that result in structures with a vast surface area, high porosity, and remarkable stability. For example, the BCT framework and non-zeotype topological net which is 4-connected, show higher photo-physical stability and excellent photo-electric sensitivity and have applications in photoinduced catalysis and light harvesting^40^. The 3D coordination polymers with BCT topology enhance the initial zeolite topologies and show remarkable improvements in adsorption, ion exchange and thermal stability^41^. MOFs with network topology of zeolite BCT and one-dimensional hydrophilic tunnel has high selectivity for different absorbates such as CO$_{2}$, H$_{2}$O, etc^42,43^. Anionic luminous MOF with a zeolite BCT architecture is employed to selectively capture and segregate cationic colors^44^. Thus, zeolite like MOFs with BCT and DFT topologies have tremendous chemical, biological, industrial and environmental applications.

**Figure 1:**
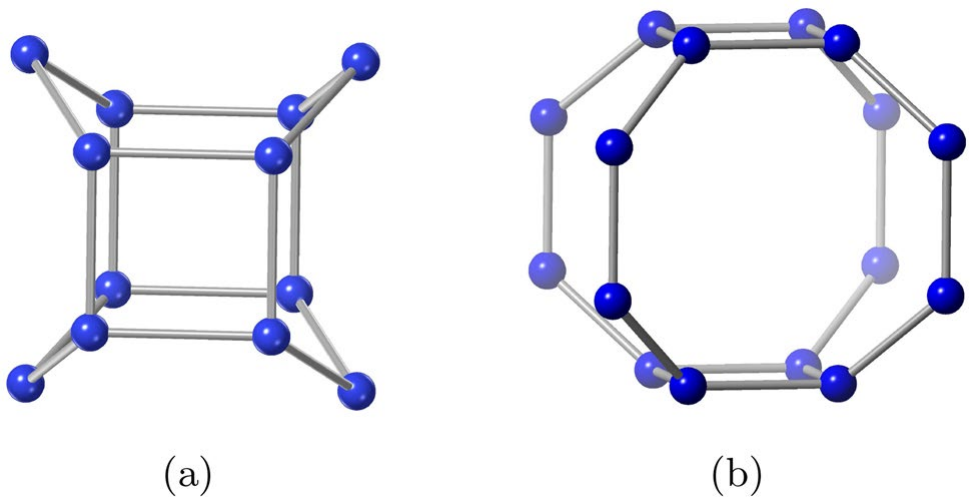
(**a**) Building cage of BCT. (**b**) Building cage of DFT.

**Figure 2:**
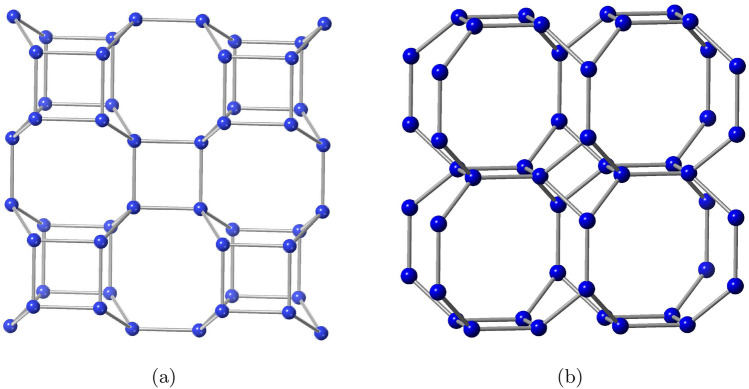
(**a**) Square arrangement of zeolite BCT. (**b**) Square arrangement of zeolite DFT.

In our study, we utilize chemical graph theory to explore the structural characteristics of BCT and DFT frameworks by representing the atoms with vertices and chemical bonds with edges and deriving their associated entropy measures. Furthermore, we make a comparative study of entropies by implementing scaled measures.

## Results

We consider the zeolites BCT and DFT materials and implement the edge partition technique for the degree measures and develop refined edge partition for the degree-sum, which consequently derives the topological properties of BCT and DFT materials, as well as extending the procedure for determining information entropy measurements.

**Figure 3:**
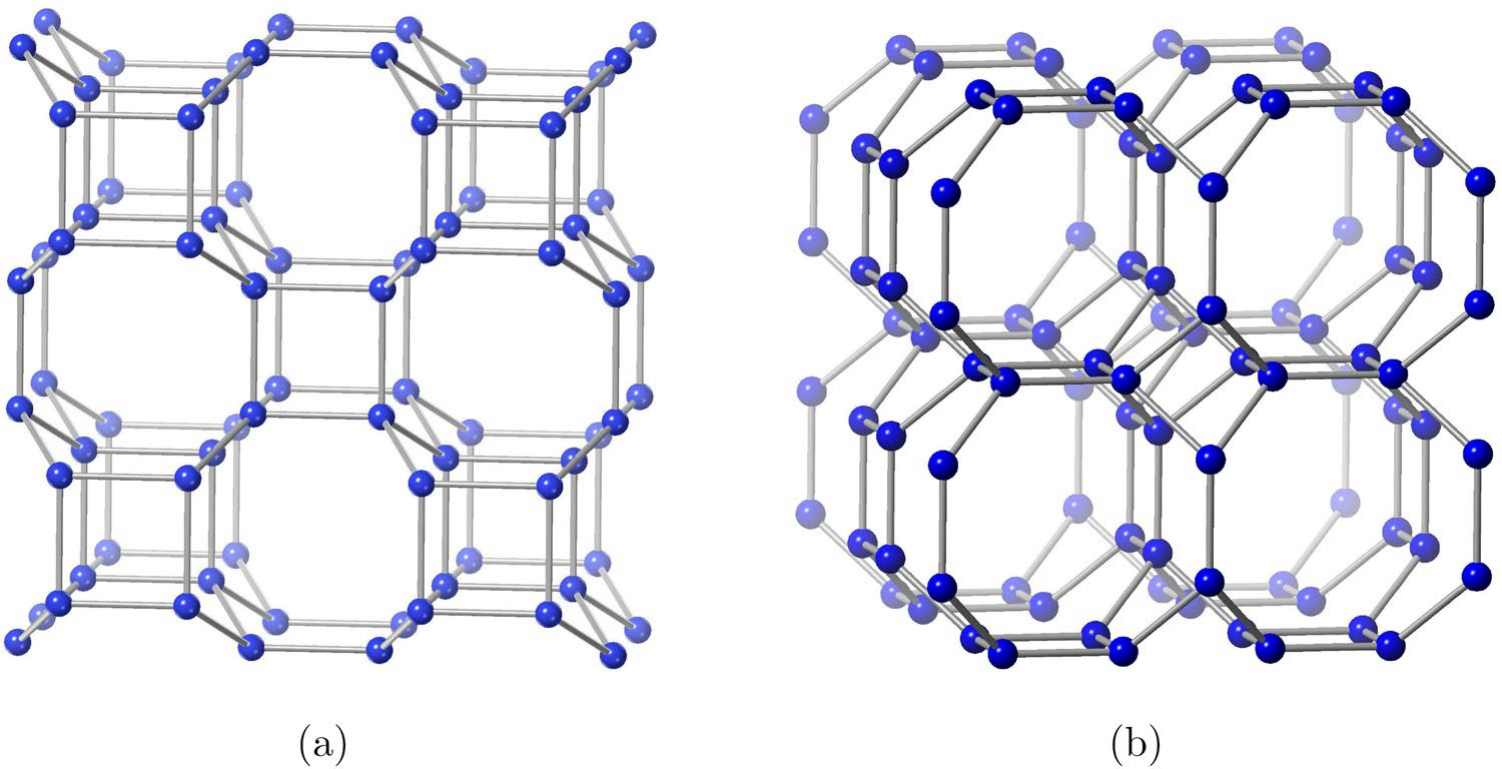
(**a**) Cubic arrangement of zeolite BCT. (**b**) Cubic arrangement of zeolite DFT.

As we see from Fig. 1, a primitive unit of BCT contains 12 vertices and 16 edges, whereas a primitive unit of DFT contains of 16 vertices and 20 edges. Zeolites BCT and DFT are built up in layers, with each layer consisting of unit cells in ($u$ $\times$ $v$) arrangement as shown in Fig. 2. Multiple layers are created from the single layer by ($u$ $\times$ $v$ $\times$ $w$) using the interlayer bridges as shown in Fig. 3. This pattern’s built-in structure is depicted by $\text {BCT}(u,v,w)$ and $\text {DFT}(u,v,w)$. Multi-layer unit cells comprise of vertices and edges which are counted as $|V(\text {BCT}(u,v,w))|=4uv(2w+1)$ and $|E(\text {BCT}(u,v,w))|=2[8uvw+(2uv-vw-wu)]$ and $|V(\text {DFT}(u,v,w))|=4w(2uv+(u+v))$ and $|E(\text {DFT}(u,v,w))|=2[8uvw-(uv-2vw-2wu)-u]$.

### Zeolite BCT: degree based topological indices

The zeolite BCT partition has five types of edge classes based on the degrees of the vertex ends on the edges of $\text {BCT}(u,v,w)$ and Table 1 shows the class cardinality.

**Table 1:** Partitions of edges in zeolite BCT: degree form.

S. no.	Class	Number of appearance
1	$d_{(2,3)}$	8
2	$d_{(2,4)}$	$8(w-1)$
3	$d_{(3,3)}$	$2[(4uv+vw+wu)+2(2u+2v-w)-8]$
4	$d_{(3,4)}$	$8[(uv+vw+wu)-2(u+v+w)+3]$
5	$d_{(4,4)}$	$4[4uvw-3(uv+vw+wu)+(2u+2v+3w)-2]$

#### Theorem 1

Let *G* denote a zeolite $\text {BCT}(u,v,w)$, where $u,v,w \ge 1$.

1. $$ {M_1}^d(G) = 4[32uvw+(2uv-7vw-7wu)+2w]$$
2. $$ {M_2}^d(G) =2[128uvw-(12uv+39vw+39wu)+2(2u+2v+7w)]$$
3. $$ R^d(G) = \frac{1}{6755399441055744}[27021597764222976uvw+ 4(3337281732355669uv - 40417988172203vw - 40417988172203wu) +8(40417988172203u +40417988172203v - 104462425026536w) + 219071496363344]$$
4. $$ABC^d(G) = \frac{1}{1688849860263936}[16547281639269258uvw + 2(2658960601342489uv - 718739119185383vw - 718739119185383wu) - 8(20190785052030u + 20190785052030v -2259094509781w) - 124489796928821]$$
5. $$ H^d(G) = \frac{1}{105}[420uvw+5(41uv-vw-wu)+5(2u+2v-5w)+6]$$
6. $$ SC^d(G) = \frac{1}{1847179534663680}[10449225400237596uvw+ (3781289962452752uv-743357360784935vw-743357360784935wu)+2(43391983903825u+43391983903825v-158670615479038w)+41506161719616]$$
7. $$ HM^d(G) = 8[128uvw-(11uv+38vw+38wu)+2(u+v+8w)]$$
8. $$GA^d(G) = \frac{1}{615726511554560}[9851624184872960uvw + 6(402063926833831uv-213662584720729vw-213662584720729wu) +4(25261242607627u+ 25261242607627v-45166736030649w)+30622844591065]$$
9. $$ IRR^d(G) = 8[(uv+vw+wu)-2(u+v)+2]$$
10. $$ \sigma ^d(G) = 8[(uv+vw+wu)-2(u+v-w)]$$
11. $$ F^d(G) = 4[128uvw-(10uv+37vw+37wu)+18]$$
12. $$ SO^d(G) =\frac{1}{140737488355328}[12738103345051546uvw + (852710779818790uv - 2729880785976957vw - 2729880785976957wu) -2( 56579320753069u + 56579320753069v - 470680759143416w) - 9818188635438]$$
13. $$ SDD^d(G) = \frac{2}{3}[48uvw+(13uv-5vw-5wu)-2(u+v-2w)-1]$$
14. $$ AZ^d(G) =\frac{1}{108000}[32768000uvw-3(930188uv+3390563vw+3390563wu)+2(1168814u+1168814v+1339689w)- 235192]$$

#### Proof

For the topological index function $\zeta ^d$, we obtain the desired expressions by using classes in the edge degree partition of Table 1 through the equation $\zeta ^d{(G)}=d_{(2,3)} \ \zeta ^d(2,3)+d_{(2,4)} \ \zeta ^d(2,4)+d_{(3,3)} \ \zeta ^d(3,3)+d_{(3,4)} \ \zeta ^d(3,4)+d_{(4,4)} \ \zeta ^d(4,4)$. $\square$

### Zeolite BCT: degree-sum based topological indices

We calculate degree-sum topological descriptors using the same method as before. Here, we can see that the refined degree-sum partitions for $w\ge 2$ and $w=1$ are not the same. The edge partition of degree-sum has seventeen classes of edges for $w\ge 2$ and seven for $w=1$. Table 2 shows class cardinality.

**Table 2:** Partitions of edges in zeolite BCT: degree-sum form.

S. no.	Class	Number of appearance for $w\ge 2$	$w=1$
1	${d_s}_{(6,8)}$	–	8
2	${d_s}_{(7,8)}$	8	–
3	${d_s}_{(7,12)}$	8	–
4	${d_s}_{(8,9)}$	16	16
5	${d_s}_{(8,12)}$	$8(w-2)$	–
6	${d_s}_{(9,9)}$	$4[(u+v)-4]$	$2[7(u+v)-18]$
7	${d_s}_{(9,10)}$	$16[(u+v)-2]$	$8[(u+v)-2]$
8	${d_s}_{(10,10)}$	$8[uv-(u+v)+1]$	$4[2uv-3(u+v)+4]$
9	${d_s}_{(10,14)}$	$8[(u+v)-2]$	$8[uv-(u+v)+1]$
10	${d_s}_{(10,15)}$	$8[uv-(u+v)+1]$	–
11	${d_s}_{(11,11)}$	$2[(vw+wu)-2(u+v+w)+4]$	–
12	${d_s}_{(11,14)}$	$8[(vw+wu)-2(u+v+w)+4]$	–
13	${d_s}_{(12,14)}$	$8(w-1)$	–
14	${d_s}_{(14,14)}$	$2[(vw+wu)-(u+v+4w)+4]$	$4[uv-(u+v)+1]$
15	${d_s}_{(14,16)}$	$4[(vw+wu)-(u+v+2w)+2]$	–
16	${d_s}_{(15,15)}$	$8[uv-(u+v)+1]$	–
17	${d_s}_{(15,16)}$	$8[uv-(u+v)+1]$	–
18	${d_s}_{(16,16)}$	$2[8uvw-(14uv+9vw+9wu)+5(3u+3v+2w)-16]$	–

Hence, we evaluate degree-sum based topological descriptors for zeolite $\text {BCT}(u,v,w)$ for the general case $w\ge 2$ and the particular case $w=1$ can be dealt in a similar way.

#### Theorem 2

Let *G* denote a zeolite $\text {BCT}(u,v,w)$, where $u,v \ge 1$ and $w\ge 2$,

1. $$ {M_1}^d(G) = 4[128uvw-(12uv+39vw+39wu)+2(2u+2v+7w)]$$
2. $$ {M_2}^d(G) =2[2048uvw-(724uv+923vw+923wu)+2(152u+152v+231w)- 120]$$
3. $$ R^d(G) = \frac{1}{30478462321950720}[30478462321950720uvw+ 7(278299883841392uv + 485891729614741vw + 485891729614741wu) +(3539039119208179u -9617167262945548v - 2291802998854154w) - 335478298794768]$$
4. $$ABC^d(G) = \frac{1}{7619615580487680}[41734353329509232uvw + (19388978247910540uv - 872029067767873vw - 872029067767873wu) +2 (345591400218920u + 345591400218920v - 828177260788439w) - 154236230512704]$$
5. $$ H^d(G) = \frac{1}{18041423400}[18041423400uvw+12597(1059058uv+152303vw+152303wu)+17(135521971u+135521971v-88099086w)- 402231920]$$
6. $$ SC^d(G) = \frac{1}{4747997762027520}[13429365658352844uvw+ (6345902302016824uv- 224665123195633vw- 224665123195633wu)+(486197048551859u+486197048551859v- 626435987712023w)- 171800053098336]$$
7. $$ HM^d(G) = 16[1024uvw-(349uv+456vw+456wu)+46(3u+3v+5w)- 49]$$
8. $$GA^d(G) = \frac{1}{616623085977600}[9865969375641600uvw + (2364258556376573uv- 1274379651427311vw- 1274379651427311wu) +(96343104076105u+ 96343104076105v- 32015854281344w)- 78435422219796]$$
9. $$ IRR^d(G) = 8[2(3uv+2vw+2wu)-(7u+7v+2w)+6]$$
10. $$ \sigma ^d(G) = 8[(26uv+11vw+11wu)-2(14u+14v+w)+22]$$
11. $$ F^d(G) = 4[2048uvw-(672uv+901vw+901wu)+2(124u+124v+229w)-76]$$
12. $$ SO^d(G) =\frac{1}{70368744177664}[25476206690103092uvw -28 (78031937749928uv + 274201137619735vw +274201137619735wu) +(589301787989786u + 589301787989786v + 2805400739061925w) + 158405138053204]$$
13. $$ SDD^d(G) = \frac{1}{6930}[221760uvw+3(21637uv-7995vw-7995wu)-2(4439u+4439v-1545w)+ 7515]$$
14. $$ AZ^d(G) =\frac{1}{474989023199232}[4722366482869645152uvw-512(4751865619046747uv+5042769299074674vw+5042769299074674wu)+512(2529098571771281u+2529098571771281v+2943986606676985w)- 689574197346513904]$$

### Zeolite DFT: degree based topological indices

The zeolite DFT partition has six types of edge classes based on degrees of $\text {DFT}(u,v,w)$ and Table 3 lists the cardinality of these classes.

**Table 3:** Partitions of edges in zeolite DFT: degree form.

S. no.	Class	Number of appearance
1	$d_{(2,2)}$	4*u*
2	$d_{(2,3)}$	8
3	$d_{(2,4)}$	$8(u-1)$
4	$d_{(3,3)}$	$2[(uv+4vw+4wu)-(5u-4v-4w)-8]$
5	$d_{(3,4)}$	$8[(uv+vw+wu)-2(u+v+w)+3]$
6	$d_{(4,4)}$	$4[4uvw-3(uv+vw+wu)+(3u+2v+2w)-2]$

#### Theorem 3

For $u,v,w \ge 1$, let *G* be the zeolite $\text {DFT}(u,v,w)$.

1. $$ {M_1}^d(G) = 4[32uvw-7(uv-2vw-2wu)-3u]$$
2. $$ {M_2}^d(G) =2[128uvw-(39uv+12vw+12wu)-(5u-4v-4w)]$$
3. $$ R^d(G) = \frac{1}{1688849860263936}[6755399441055744uvw - (40417988172203uv - 3337281732355669vw - 3337281732355669wu) -2(104462425026536u -40417988172203v -40417988172203w) + 54767874090834]$$
4. $$ABC^d(G) = \frac{1}{844424930131968}[8273640819634629uvw -(718739119185383uv - 2658960601342489vw - 2658960601342489wu) - 2(490134482645791u + 40381570104060v +40381570104060w) - 62244898464410]$$
5. $$ H^d(G) = \frac{1}{105}[420uvw-5(uv-41vw-41wu)-5(5u-2v-2w)+6]$$
6. $$ SC^d(G) = \frac{1}{1847179534663680}[10449225400237596uvw- (743357360784935uv-3781289962452752vw-3781289962452752wu)-2(573814742434202u-43391983903825v-43391983903825w)+41506161719616]$$
7. $$ HM^d(G) = 8[128uvw-(38uv+11vw+11wu)-(3u-2v-2w)]$$
8. $$GA^d(G) = \frac{1}{307863255777280}[4925812092436480uvw -3(213662584720729uv-402063926833831vw-402063926833831wu) -2(353029991807929u-25261242607627v-25261242607627w)+15311422295532]$$
9. $$ IRR^d(G) = 8[(uv+vw+wu)-2(v+w)+2]$$
10. $$ \sigma ^d(G) = 8[(uv+vw+wu)+2(u-v-w)]$$
11. $$ F^d(G) = 4[128uvw-(37uv+10vw+10wu)-u]$$
12. $$ SO^d(G) =\frac{1}{140737488355328}[12738103345051546uvw - (2729880785976957uv - 852710779818790vw - 852710779818790wu) -2( 524483564688736u + 56579320753069v + 56579320753069w) - 9818188635438]$$
13. $$ SDD^d(G) = \frac{2}{3}[48uvw-(5uv-13vw-13wu)-2(u+v+w)-1]$$
14. $$ AZ^d(G) =\frac{1}{108000}[32768000uvw-3(3390563uv+930188vw+930188wu)-(1245747u-2337628v-2337628w)- 235192]$$

#### Proof

For the topological index function $\zeta ^d$, we obtain the desired expression by using classes in the edge degree partition of Table 3 through the equation $\zeta ^d{(G)}=d_{(2,2)} \ \zeta ^d(2,2)+d_{(2,3)} \ \zeta ^d(2,3)+d_{(2,4)} \ \zeta ^d(2,4)+d_{(3,3)} \ \zeta ^d(3,3)+d_{(3,4)} \ \zeta ^d(3,4)+d_{(4,4)} \ \zeta ^d(4,4)$. $\square$

### Zeolite DFT: degree-sum based topological indices

Table 4 shows the refined degree-sum partition of zeolite DFT in which the edges partitioned into 16 types of classes for $w\ge 2$ and thirteen for $w=1$. Then, we compute the degree-sum topological descriptors using the same method for degree.

#### Theorem 4

For $u,v \ge 1$ and $w\ge 2$, let *G* be the zeolite $\text {DFT}(u,v,w)$.

1. $$ {M_1}^d(G) = 4[128uvw-(39uv+12vw+12wu)-(5u-4v-4w)]$$
2. $$ {M_2}^d(G) =2[2048uvw-(923uv+700vw+700wu)+(215u+298v+320w)- 98]$$
3. $$ R^d(G) = \frac{1}{30478462321950720}[12381875318292480uvw+ (3401242107303187uv + 23201479170288107vw + 23201479170288107wu) +(1038372196153755u +1889170820978314v + 2161567386220469w) + 2668102493342356]$$
4. $$ABC^d(G) = \frac{1}{7619615580487680}[41734353329509232uvw - (872029067767873uv - 19400901886739074vw - 194009018867390741wu) - (3525478178361867u - 736940656494876v - 771347015196364w) + 196903941721256]$$
5. $$ H^d(G) = \frac{1}{581981400}[581981400uvw+(61889061uv+437178885vw+437178885wu)+(16117054u+79200654v+92888510w)+46558751]$$
6. $$ SC^d(G) = \frac{1}{5207481416417280}[14728981689806346uvw -21 (11733662348159uv-334302513432530vw-334302513432530wu)-(1436841745893208u-563001956568285v-668379780396700w)+173054749651196]$$
7. $$ HM^d(G) = 16[1024uvw-(456uv+341vw+341wu)+(113u+136v+143w)- 42]$$
8. $$GA^d(G) = \frac{1}{676296287846400}[10820740605542400uvw -(1397706714468663uv-2623493692919504vw-2623493692919504wu) -(1551242003521687u- 105746902190190v-148385348247770w)- 34160231501508]$$
9. $$ IRR^d(G) = 8[(5uv+5vw+5wu)-(u+7v+8w)+8]$$
10. $$ \sigma ^d(G) = 8[(11uv+18vw+18wu)+(11u-26v-34w)+14]$$
11. $$ F^d(G) = 4[2048uvw-(901uv+664vw+664wu)+3(79u+82v+84w)- 70]$$
12. $$ SO^d(G) =\frac{1}{140737488355328}[50952413380206184uvw -4 (3838815926676290uv + 1121680261022383vw +1121680261022383wu) -(1613379445377556u - 1193539352812283v - 1054000175616380w) + 165515603305936]$$
13. $$ SDD^d(G) = \frac{1}{6930}[221760uvw-9(2665uv-6919vw-6919wu)-2(4623u+4415v+6215w)+1251]$$
14. $$ AZ^d(G) =\frac{1}{2199023255552000}[21862807791063172000uvw-375(31875282483039912uv+28421134094158000vw+28421134094158000wu)+(5027711667562645823u+5763769618488125000v+6222315416680717500w)- 2911857434351704896]$$

**Table 4:** Partitions of edges in zeolite DFT: degree-sum form.

S. no.	Class	Number of appearance for $w\ge 2$	$w=1$
1	${d_s}_{(5,6)}$	8	8
2	${d_s}_{(5,8)}$	8	8
3	${d_s}_{(6,6)}$	$4(u-2)$	$4(u-2)$
4	${d_s}_{(6,12)}$	$8(u-1)$	$8(u-1)$
5	${d_s}_{(8,8)}$	–	4
6	${d_s}_{(8,9)}$	16	8
7	${d_s}_{(9,9)}$	$4[(3v+4w)-9]$	$4(4v-5)$
8	${d_s}_{(9,10)}$	$8[(v+2w)-3]$	–
9	${d_s}_{(9,11)}$	$4(v-1)$	$4(v-1)$
10	${d_s}_{(10,10)}$	$4[2(vw+wu)-(2u+3v+6w)+7]$	–
11	${d_s}_{(10,14)}$	$8[(vw+wu)-(u+v+2w)+2]$	–
12	${d_s}_{(11,11)}$	$2[uv-(u+2v)+2]$	$2[uv-(u+2v)+2]$
13	${d_s}_{(11,14)}$	$8[uv-(u+v)+1]$	$8[uv-(u+v)+1]$
14	${d_s}_{(12,12)}$	–	$2(u-1)$
15	${d_s}_{(12,14)}$	$8(u-1)$	$4(u-1)$
16	${d_s}_{(14,14)}$	$2[(uv+vw+wu)-2(2u+v+w)+5]$	$2[2uv-(3u+2v)+3]$
17	${d_s}_{(14,16)}$	$4[(uv+vw+wu)-2(u+v+w)+3]$	–
18	${d_s}_{(16,16)}$	$2[8uvw-9(uv+vw+wu)+10(u+v+w)-11]$	–

**Figure 4:**
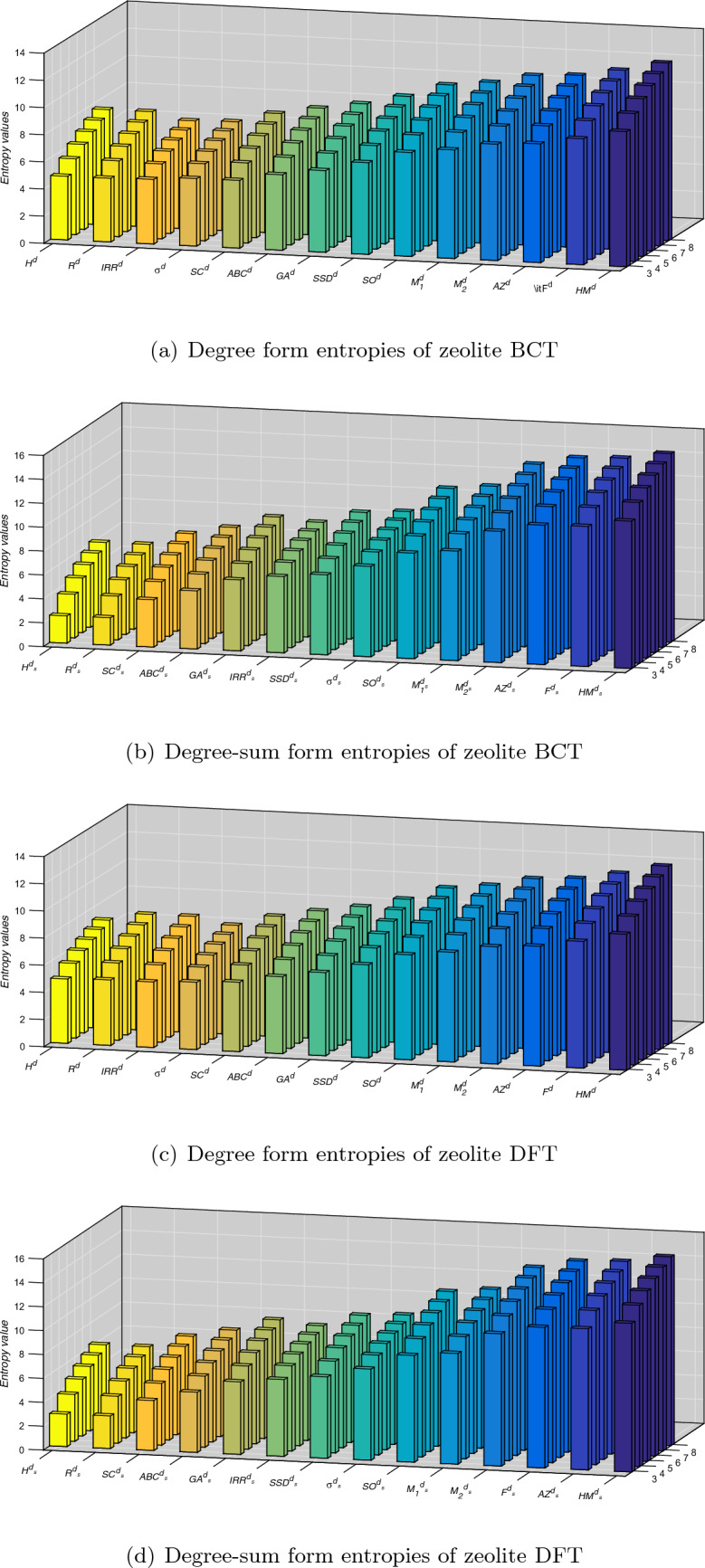
Comparison of degree and degree-sum form entropies in cubic zeolite BCT & DFT.

**Table 5:** Entropy values for cubic zeolite BCT: degree form.

$I^{*}(\zeta ^{d})$	$u=3$	$u=4$	$u=5$	$u=6$	$u=7$	$u=8$
$M_1^d$	7.9962	8.9058	9.5994	10.1613	10.6340	11.0420
$M_2^d$	8.5726	9.5132	10.2246	10.7982	11.2791	11.6932
$R^d$	4.6896	5.5870	6.2651	6.8127	7.2730	7.6707
$ABC^d$	5.5583	6.4437	7.1205	7.6698	8.1328	8.5331
$H^d$	4.6835	5.5826	6.2616	6.8097	7.2705	7.6685
$SC^d$	4.9902	5.8906	6.5730	7.1246	7.5884	7.9891
$HM^d$	9.9553	10.9010	11.6135	12.1872	12.6680	13.0820
$GA^d$	6.0185	6.9060	7.5857	8.1378	8.6032	9.0055
$IRR^d$	4.7592	5.5185	6.0658	6.4938	6.8454	7.1439
$\sigma ^d$	4.9750	5.6811	6.1961	6.6026	6.9388	7.2257
$F^d$	9.2748	10.2155	10.9259	11.4985	11.9785	12.3919
$SO^d$	7.6545	8.5625	9.2554	9.8169	10.2892	10.6970
$SSD^d$	6.7499	7.6251	8.2982	8.8464	9.3091	9.7096
$AZ^d$	8.7477	9.6857	10.3961	10.9691	11.4496	11.8636

**Table 6:** Entropy values for cubic zeolite BCT: degree-sum form.

$I^{*}(\zeta ^{d_{s}})$	$u=3$	$u=4$	$u=5$	$u=6$	$u=7$	$u=8$
${M_1}^{d_{s}}$	9.1693	10.1679	10.8988	11.4806	11.9655	12.3819
${M_2}^{d_{s}}$	10.9866	12.0790	12.8523	13.4587	13.9599	14.3880
$R^{d_{s}}$	2.2947	3.6569	4.5721	5.2450	5.7761	6.2162
$ABC^{d_{s}}$	4.8641	5.8153	6.5243	7.0891	7.5598	7.9640
$H^{d_{s}}$	2.2828	3.6495	4.5669	5.2409	5.7728	6.2134
$SC^{d_{s}}$	3.9607	5.0231	5.7865	6.3797	6.8665	7.2806
$HM^{d_{s}}$	12.3323	13.4529	14.2345	14.8441	15.3466	15.7753
$GA^{d_{s}}$	5.9463	6.8670	7.5629	8.1233	8.5934	8.9986
$IRR^{d_{s}}$	6.3855	7.1197	7.6532	8.0725	8.4181	8.7121
$\sigma ^{d_{s}}$	7.5664	8.3271	8.8698	9.2934	9.6413	9.9369
$F^{d_{s}}$	11.6689	12.7737	13.5499	14.1569	14.6581	15.0859
$SO^{d_{s}}$	8.8376	9.8286	10.5568	11.1373	11.6216	12.0375
$SSD^{d_{s}}$	6.6948	7.5981	8.2835	8.8376	9.3036	9.7060
$AZ^{d_{s}}$	11.7215	12.8640	13.6609	14.2818	14.7928	15.2280

**Table 7:** Entropy values for cubic zeolite DFT: degree form.

$I^{*}(\zeta ^{d})$	$u=3$	$u=4$	$u=5$	$u=6$	$u=7$	$u=8$
$M_1^d$	8.0789	8.9698	9.6516	10.2054	10.6721	11.0756
$M_2^d$	8.6392	9.5638	10.2656	10.8325	11.3086	11.7191
$R^d$	4.8245	5.6913	6.3509	6.8857	7.3366	7.7271
$ABC^d$	5.6681	6.5299	7.1916	7.7304	8.1855	8.5799
$H^d$	4.8196	5.6875	6.3477	6.8830	7.3343	7.7251
$SC^d$	5.1084	5.9825	6.6485	7.1888	7.6443	8.0386
$HM^d$	10.0215	10.9513	11.6542	12.2214	12.6974	13.1078
$GA^d$	6.1232	6.9877	7.6528	8.1948	8.6526	9.0493
$IRR^d$	4.7391	5.5073	6.0586	6.4888	6.8416	7.1409
$\sigma ^d$	4.9587	5.6716	6.1898	6.5980	6.9353	7.2230
$F^d$	9.3405	10.2656	10.9665	11.5325	12.0078	12.4177
$SO^d$	7.7369	8.6263	9.3075	9.8609	10.3273	10.7306
$SSD^d$	6.8522	7.7055	8.3645	8.9028	9.3581	9.7530
$AZ^d$	8.8222	9.7419	10.4411	11.0067	11.4819	11.8918

**Table 8:** Entropy values for cubic zeolite DFT: degree-sum form.

$I^{*}(\zeta ^{d_{s}})$	$u=3$	$u=4$	$u=5$	$u=6$	$u=7$	$u=8$
${M_1}^{d_{s}}$	9.2648	10.2297	10.9451	11.5179	11.9969	12.4090
${M_2}^{d_{s}}$	11.0798	12.1291	12.8861	13.4843	13.9806	14.4053
$R^{d_{s}}$	2.7294	3.9327	4.7637	5.3892	5.8913	6.3120
$ABC^{d_{s}}$	5.0386	5.9418	6.6225	7.1695	7.6280	8.0233
$H^{d_{s}}$	2.7162	3.9246	4.7579	5.3848	5.8876	6.3088
$SC^{d_{s}}$	4.1835	5.1760	5.9002	6.4701	6.9416	7.3449
$HM^{d_{s}}$	12.4383	13.5077	14.2705	14.8708	15.3679	15.7930
$GA^{d_{s}}$	6.0688	6.9581	7.6353	8.1835	8.6449	9.0437
$IRR^{d_{s}}$	6.4252	7.1407	7.6663	8.0814	8.4245	8.7170
$\sigma ^{d_{s}}$	7.6298	8.3622	8.8907	9.3058	9.6481	9.9397
$F^{d_{s}}$	11.7706	12.8270	13.5851	14.1832	14.6792	15.1035
$SO^{d_{s}}$	8.9308	9.8896	10.6028	11.1745	11.6528	12.0646
$SSD^{d_{s}}$	6.8161	7.6884	8.3556	8.8978	9.3553	9.7514
$AZ^{d_{s}}$	11.8166	12.9101	13.6902	14.3029	14.8094	15.2417

## Comparative analysis between zeolites BCT and DFT

We employ the degree and degree-sum descriptors from the previous sections in Eq. (2) to derive entropies through a modified Shannon’s approach method for zeolites BCT and DFT.

The extensive formulas for each index’s entropy would take up a lot of space as expressions. As a result, we present a tabular representation of numerical values of entropy measures and comparison charts. It can be seen in the Fig. 4 and in Tables 5, 6, 7 and 8 that indicates the hyper-Zagreb index has higher entropy for zeolites BCT and DFT.

As we have computed the bond-additive entropy values for zeolites BCT and DFT, it is not directly feasible to make a comparative analysis between them as they have different numbers of bonds. Hence, it is necessary to consider the scaled entropy measures to make a relatively fair comparison between BCT and DFT. Therefore, we compute the scaled entropy measures for zeolites BCT and DFT by dividing the total entropy values with the total number of bonds in the considered zeolites in order to analyze the stability and bond energies between BCT and DFT. Though, we have found the degree and degree-sum types of entropies in the preceding part, we here perform the comparative analysis based on the degree, and a similar process can be performed entropies.

**Table 9:** Scaled entropies of zeolites $\text {BCT}(u,v,w)$ and $\text {DFT}(u,v,w)$.

$({{\zeta }}^{d})$	BCT(3,3,3)	DFT(3,3,3)	BCT(4,4,4)	DFT(4,4,4)	BCT(5,5,5)	DFT(5,5,5)	BCT(6,6,6)	DFT(6,6,6)
${{M_1}}^{{d}}$	0.0185	0.0168	0.0087	0.0081	0.0048	0.0045	0.0029	0.0028
${{M_2}}^{{d}}$	0.0198	0.0180	0.0093	0.0086	0.0051	0.0048	0.0031	0.0030
${{R}}^{{d}}$	0.0109	0.0101	0.0055	0.0051	0.0031	0.0030	0.0020	0.0019
${{ABC}}^{{d}}$	0.0129	0.0118	0.0063	0.0059	0.0036	0.0034	0.0022	0.0021
${{H}}^{{d}}$	0.0108	0.0100	0.0055	0.0051	0.0031	0.0030	0.0020	0.0019
${{SC}}^{{d}}$	0.0116	0.0106	0.0058	0.0054	0.0033	0.0031	0.0021	0.0020
${{HM}}^{{d}}$	0.0230	0.0209	0.0106	0.0098	0.0058	0.0054	0.0035	0.0033
${{GA}}^{{d}}$	0.0139	0.0128	0.0067	0.0063	0.0038	0.0036	0.0024	0.0022
${{IRR}}^{{d}}$	0.0110	0.0099	0.0054	0.0050	0.0030	0.0028	0.0019	0.0018
${{{\sigma }}}^{{d}}$	0.0115	0.0103	0.0055	0.0051	0.0031	0.0029	0.0019	0.0018
${{F}}^{{d}}$	0.0215	0.0195	0.0100	0.0092	0.0055	0.0051	0.0033	0.0032
${{SO}}^{{d}}$	0.0177	0.0161	0.0084	0.0078	0.0046	0.0043	0.0028	0.0027
${{SSD}}^{{d}}$	0.0156	0.0143	0.0074	0.0069	0.0041	0.0039	0.0026	0.0024
${{AZ}}^{{d}}$	0.0202	0.0184	0.0095	0.0088	0.0052	0.0049	0.0032	0.0030

**Figure 5:**
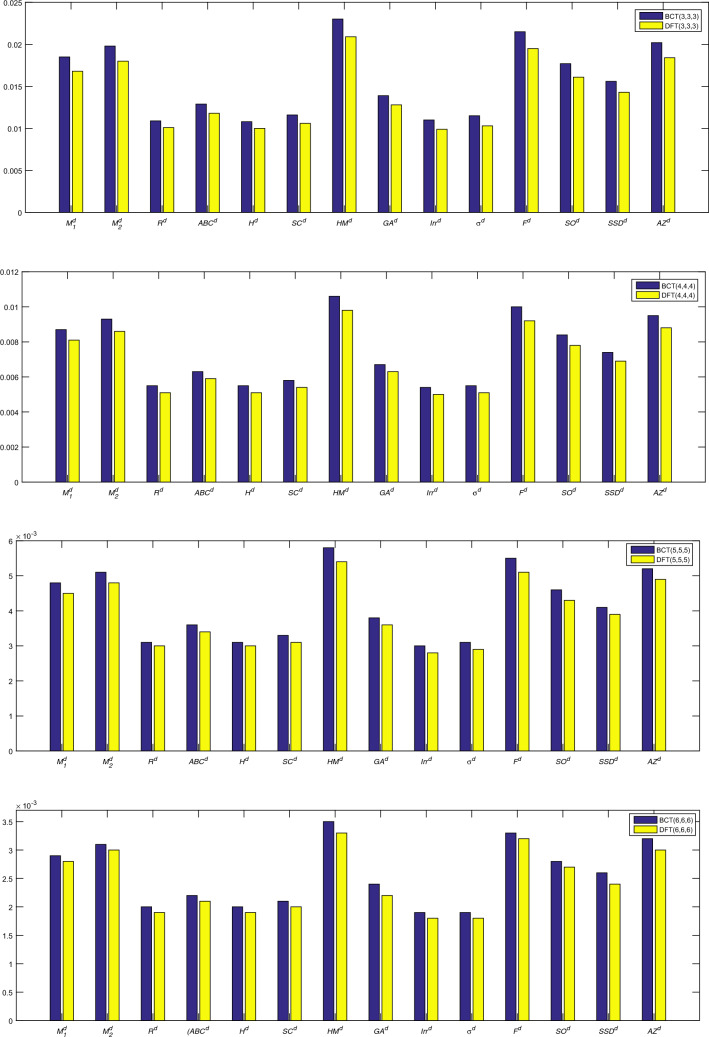
Comparison of scaled entropies of zeolites BCT and DFT.

Table 9 and Fig. 5 show that the zeolite BCT consistently displays higher entropy relative to the zeolite DFT across all cases as described by the set of parameters (*u*, *v*, *w*). As we mentioned earlier, entropy is a measure of the disorder or randomness in a system; a higher value of entropy implies high randomness in your system. In other words, it is difficult to predict the state of atoms or molecules in it with higher entropy compared to lower entropy. The experimental results of a few mechanical properties of zeolites materials^45^ show that the yield strength of 46.1 megapascals for zeolite DFT is higher when compared to 32.0 megapascals for zeolite BCT, and similarly, the elastic modulus of zeolite DFT is 0.95 gigapascals higher when compared to 0.81 gigapascals for zeolite BCT, which demonstrates that our study is consistent with experimental results. If one can pair the topological differences across structures with quantum chemical factors like hardness, electrophilicity indices, and relative energy, that will give new results pertaining to relative free energies, stabilities, and phase transition occurrences. It is believed that the current study will influence future work in quantum structural research, including work on the potential use of machine learning to develop hybrid approaches.

## Conclusion

We have discussed the zeolites BCT and DFT through degree measures by employing edge partition technique and developed refined edge partition via degree-sum measures to derive their topological properties. We have used such properties to obtain the entropy values by modified Shannon’s approach and the results show that the hyper-Zagreb index has high entropy values when compared with other descriptors. Further, we have analyzed the stability and bond energies of zeolites by scaled entropy approach between zeolites BCT and DFT. The study shows that the zeolite BCT consistently displays higher entropy relative to the zeolite DFT across all cases. This study paves the way for further study of the surface area, enthalpy formation, pressure, stability, energy and many other properties of chemical compounds that will help in the synthesis process of new frameworks.

## Data Availability

All data used for this study are contained in the article.
